# Analysis of the prevalence of dyslipidemia in early-onset schizophrenia patients and its correlation with clinical characteristics

**DOI:** 10.3389/fpsyt.2026.1794475

**Published:** 2026-04-07

**Authors:** Kehuang Li, Zhiwei Liu, Liang Sun, Jingjing Zhang, Xinglong Yin, Zhaokun Zhang, Changjie Cao

**Affiliations:** 1 Department of Pharmacy, The Third People’s Hospital of Fuyang, Fuyang, China; 2 Department of Psychiatry, The Third People’s Hospital of Fuyang, Fuyang, China; 3 Department of Psychiatry, Fuyang Mental Health Center, Fuyang, China

**Keywords:** antipsychotic drugs, body roundness index, dyslipidemia, early-onset schizophrenia, prevalence, TyG index

## Abstract

**Objective:**

To analyze the prevalence of dyslipidemia and related influencing factors in patients with early-onset schizophrenia (EOS).

**Methods:**

We recruited 289 pediatric and adolescent EOS patients from October 2021 to June 2024 in the Third People’s Hospital of Fuyang. Researchers gathered comprehensive demographic and clinical records. Utilizing the 2023 Chinese Guidelines for Lipid Management, they calculated dyslipidemia prevalence and the incidence of irregularities in total cholesterol, triglycerides, LDL cholesterol, HDL cholesterol, and non-HDL cholesterol. Subsequently, differences in dyslipidemia among different genders, body mass index, and antipsychotic medication groups were analyzed. Finally, independent influencing factors of dyslipidemia in EOS patients were explored.

**Results:**

The overall prevalence of dyslipidemia was 24.9% (72/289), with abnormal rates of TG, TC, HDL-C, LDL-C, and non-HDL-C being 15.9%, 6.6%, 6.6%, 4.2%, and 7.3%, respectively. Male patients, those who were overweight or obese, and those taking two antipsychotic drugs had significantly higher rates of dyslipidemia. Regression analysis showed that male gender (*OR* = 2.04, *P* = 0.016), overweight/obesity (*OR* = 4.55, *P* < 0.001), body roundness index (*OR* = 1.53, *P* = 0.005), and the use of two antipsychotic drugs (*OR* = 1.90, *P* = 0.030) were risk factors for dyslipidemia in EOS patients.

**Conclusion:**

The prevalence of dyslipidemia in EOS patients is relatively high. When monitoring lipid levels in clinical practice, particular attention should be paid to male patients, those who are overweight or obese, and those receiving combined drug therapy.

## 1 Introduction

Economic and social progress in China has led to altered nutritional intake, decreased exercise, and increased lifestyle-related risk factors. Consequently, dyslipidemia is becoming more common in the nation’s youth. A recent comprehensive meta-analysis reveals an overall dyslipidemia prevalence of 19% within this demographic. Detection rates for various lipid abnormalities, including elevated total cholesterol (TC), triglycerides (TG), and low-density lipoprotein cholesterol (LDL-C), as well as reduced high-density lipoprotein cholesterol (HDL-C), are all rising. This trend presents a pressing public health concern (1). Literature reports that early diagnosis and treatment of schizophrenia are crucial for a good prognosis, and the early use of antipsychotic drugs has become the key to treatment (2). However, dyslipidemia, overweight, and obesity related to drug treatment have become difficult points in clinical practice (3). Dyslipidemia poses a significant risk for the development of cardiovascular and cerebrovascular disorders, necessitating prompt intervention and therapeutic strategies.

Early-onset schizophrenia (EOS), defined by its manifestation prior to adulthood, is distinguished by a premature onset, therapeutic challenges, and an unfavorable clinical trajectory. Studies have shown that first-admission schizophrenia patients exhibit significant abnormalities in TG and HDL-C levels after receiving drug treatment, and these abnormalities are more pronounced in patients who have been on long-term medication (4). Historical studies reveal that individuals diagnosed with schizophrenia face significantly higher health risks, exhibiting a mortality rate 2–3 times greater and a life expectancy reduced by 10–20 years compared to the general populace (5). As one of the primary treatments for schizophrenia, antipsychotic medications improve clinical symptoms while up to 60% of patients suffer negative metabolic consequences to varying degrees (6). Furthermore, there is a robust correlation between the utilization of antipsychotics and heightened incidence of accidental fatalities among children and adolescents, particularly evident within the cohort prescribed elevated dosages (7). In recent years, the body roundness index (BRI) and triglyceride-glucose (TyG) index have emerged as important metabolic assessment tools, demonstrating significant value in the early screening and risk prediction of dyslipidemia in adolescents. Both are closely related to an increased risk of cardiovascular disease (8–10). Based on this, this study focuses on EOS patients to explore the detection rate of dyslipidemia in this group and its independent influencing factors.

## 2 Methods and subjects

### 2.1 Participants

The study cohort consisted of pediatric inpatients admitted to the Third People’s Hospital of Fuyang between October 2021 and June 2024. Eligible participants were aged 10 to 18 years and received a clinical diagnosis of schizophrenia according to DSM-5 criteria. Exclusion criteria comprised comorbid psychiatric diagnoses per DSM-5 (e.g., schizoaffective or eating disorders), concurrent intellectual disability or major neurological conditions, significant physical illnesses affecting neurological, respiratory, or immune systems, as well as pregnancy or lactation status. This investigation obtained ethical approval from the hospital’s Ethics Committee (Approval No (2021).:2021-340-11). All participating patients and their legal guardians provided written informed consent after receiving a comprehensive explanation of the study procedures.

### 2.2 Evaluation of research variables

#### 2.2.1 Demographic data and clinical characteristics

A standardized survey instrument captured demographic information for all participants, including age and sex. It also documented clinical parameters: the overall illness duration, categories of prescribed pharmacotherapy, and their dosages. Specifically, antipsychotic medication amounts were standardized into chlorpromazine equivalent units (11).

#### 2.2.2 Hematological indicators

Morning fasting venous blood samples were drawn from all participants from 6:00 to 7:00 AM. These specimens were analyzed for fasting blood glucose (FBG), total cholesterol (TC), triglycerides (TG), high-density lipoprotein cholesterol (HDL-C), low-density lipoprotein cholesterol (LDL-C), and related hematological parameters. Non-HDL-C levels were derived using the equation: non-HDL-C = TC − HDL-C. Furthermore, the triglyceride-glucose (TyG) index was computed as: ln [(TG (mg/dL) × FBG (mg/dL))/2] (12). The calculation formula for the BRI index (13) is: BRI = 364.2-365.5* $\sqrt{1-\frac{Waist\;circumference/{\left(2\pi \right)}^{2}}{{\left(0.5*Height\right)}^{2}}}$.

#### 2.2.3 Assessment of mental symptoms

The severity of patients’ psychiatric symptoms was assessed using the 30-item Positive and Negative Syndrome Scale (PANSS-30). This instrument comprises a 7-item positive symptom subscale, a 7-item negative symptom subscale, and a 16-item general psychopathology subscale (14). Scoring for each item follows a 7-point system, with higher ratings indicating greater symptom severity (14). A score of 1 indicates that the patient does not have the symptoms defined by the item, while a score of 7 (extremely severe) indicates that the patient has an extremely severe psychopathological state, with the symptoms’ behavioral manifestations completely interfering with most or all major life functions, requiring close monitoring and comprehensive assistance.

### 2.3 Diagnostic criteria for dyslipidemia

As stipulated in China’s 2023 Guidelines for Lipid Management, specific diagnostic thresholds are delineated for various lipid parameters: TC at or above 5.2 mmol/L; TG at or above 1.1 mmol/L for patients under 10 years old, and 1.5 mmol/L for those aged 10 and over; HDL-C below 1.0 mmol/L; LDL-C at or above 3.4 mmol/L; and non-HDL-C at or above 3.7 mmol/L. Meeting any one of these criteria indicates dyslipidemia (15).

### 2.4 Statistical methods

This investigation utilized suitable statistical techniques for data analysis. Categorical data were summarized using counts (n) and percentages (%), while ordinal categorical variables were further detailed with medians and interquartile ranges. Normality assessments guided the description of continuous variables, presented as either mean ± standard deviation or median with interquartile range. For group comparisons, independent t-tests were applied to normally distributed continuous data with equal variances; otherwise, the Mann-Whitney U test was employed. Categorical comparisons used χ² or Fisher’s exact tests. To identify independent determinants of dyslipidemia, variables demonstrating significance (*P* < 0.05) in univariate screening were entered into a binary logistic regression model, employing forward stepwise selection based on likelihood ratio tests. Prior to multivariate analysis, multicollinearity among independent variables was assessed using Variance Inflation Factors (VIF), with VIF < 5 indicating the absence of significant collinearity. A *P*-value below 0.05 defined statistical significance for all analyses.

## 3 Results

### 3.1 Comparison between EOS patients with and without dyslipidemia

This study enrolled 289 participants, comprising 133 men and 156 women. Dyslipidemia was identified in 24.9% of the cohort (72 cases). The prevalence rates for abnormal levels of TG, TC, HDL-C, LDL-C, and non-HDL-C were 15.9% (46 cases), 6.6% (19 cases), 6.6% (19 cases), 4.2% (12 cases), and 7.3% (21 cases), in that order. Comparative analysis revealed significant statistical disparities between the groups with and without dyslipidemia concerning gender, BMI, BMI category, waist circumference, all measured lipid parameters, the BRI, the TyG index, lipid ratios, antipsychotic medication classes, and chlorpromazine equivalents (*P* < 0.05), as detailed in **Table 1**.

**Table 1:** Comparison of general data and biological indicators on whether EOS patients are accompanied by dyslipidemia.

Variables	Dyslipidemia group (*n*=72)	Non-dyslipidemia group (*n*=217)	t/Z/*χ^2^*	*P*
Age (years)	17 (15, 18)	16 (15, 17.5)	-1.685	0.092^b^
Gender (%)				
Male	40 (55.6)	93 (42.9)	3.509	**0.031^c^**
Female	32 (44.4)	124 (57.1)		
Duration of illness (month)	12 (6, 36)	12 (6, 24)	-0.778	0.436^b^
Number of admissions	1 (1, 2)	1 (1, 2)	-0.375	0.707^b^
Hospitalization category				
First episode	18 (25.0)	71 (32.7)	1.511	0.219^c^
Recurrence	54 (75.0)	146 (67.3)		
BMI	24.2 ± 5.5	20.2 (18.3, 22.3)	-5.244	**<0.001^b^**
BMI category (%)				
Underweight	4 (5.6)	23 (10.6)	27.866	**<0.001^c^**
Normal	32 (44.4)	154 (71.0)		
Overweight / Obese	36 (50.0)	40 (18.4)		
Waist circumference (cm)	81.6 ± 15.5	72.0 (65.0, 79.8)	-3.987	**<0.001^b^**
Hematological index				
FBG (mmol/L)	5.00 ± 0.75	4.96 ± 0.60	-0.401	0.689^a^
TG (mmol/L)	1.66 (1.07, 2.01)	0.80 ± 0.26	-8.983	**<0.001^b^**
TC (mmol/L)	4.38± 0.98	3.80 ± 0.62	-5.904	**<0.001^a^**
HDL-C (mmol/L)	1.22 ± 0.35	1.44 ± 0.27	5.478	**<0.001^a^**
LDL-C (mmol/L)	2.67 ± 0.69	2.17 ± 0.45	-7.080	**<0.001^a^**
non-HDL-C (mmol/L)	3.16 ± 0.91	2.35 ± 0.60	-8.573	**<0.001^a^**
BRI	4.00 ± 0.93	3.65 ± 0.87	-4.898	**0.004^a^**
TyG index	1.34 ± 0.57	0.66 ± 0.33	-12.338	**<0.001^a^**
TG/HDL-C	1.43 (0.99, 1.85)	0.58 ± 0.24	-9.471	**<0.001^b^**
TC/HDL-C	3.77 ± 1.02	2.70 ± 0.56	-11.169	**<0.001^a^**
PANSS	96.9 ± 25.3	98.8 ± 24.1	0.278	0.781^a^
Aps (%)				
Monotherapy	34 (47.2)	137 (63.1)	6.531	**0.038^c^**
Polypharmacy	38 (52.8)	80 (36.9)		
Chlorpromazine equivalent (mg/d)	379.4 ± 204.1	285 (195, 500)	-3.567	**0.017^b^**

BMI, Body Mass Index; FBG, Fasting Blood Glucose; TG, Triglycerides; TC, Total Cholesterol; HDL-C, High-Density Lipoprotein Cholesterol; LDL-C, Low-Density Lipoprotein Cholesterol; non-HDL-C, Non-High-Density Lipoprotein Cholesterol; BRI, Body Roundness Index; TyG Index, Triglycerides-Glucose Index; PANSS, Positive and Negative Symptom Scale; Aps, Antipsychotics; a, Two Independent Samples t-test; b, Wilcoxon Rank Sum Test; c, Chi-Square Test; Bolded p value, < 0.05.

### 3.2 Comparison of the prevalence of single dyslipidemia in different groups of EOS patients

The analysis of patients divided into different genders, hospitalization categories, BMI grades, and Aps groups revealed that the abnormal rates of TG, LDL-C, and non-HDL-C in male patients were significantly higher than those in female patients, being 22.6% *vs.* 10.3%, 6.8% *vs.* 1.9%, 11.3% *vs.* 3.8%, respectively. Overweight and obese subjects exhibited markedly elevated rates of dysregulated lipid parameters compared to the normal BMI group. Specifically, abnormal TG levels were 34.2% versus 9.4%, TC 13.2% vs. 4.2%, HDL-C 11.8% vs. 4.7%, LDL-C 11.8% vs. 1.4%, and non-HDL-C 18.4% vs. 3.3%. Furthermore, patients prescribed two antipsychotic agents showed higher rates of lipid abnormalities for TG (21.2% vs. 12.6%), TC (8.5% vs. 5.3%), and HDL-C (8.5% vs. 5.3%) relative to those on a single agent. No statistically significant difference emerged between first-onset and non-first-onset patient groups, as detailed in **Table 2**.

**Table 2:** Comparison of the prevalence of abnormal single lipid indicators among EOS patients in different groups.

Variables	Groups	Elevated TG (%)	Elevated TC (%)	Decreased HDL-C (%)	Elevated LDL-C (%)	Elevated non-HDL-C (%)
Gender	Male (n=133)	22.6	8.3	7.5	6.8	11.3
	Female (n=156)	10.3	5.1	5.8	1.9	3.8
	*χ^2^*	8.116	1.154	0.358	4.232	5.885
	*P*	**0.004**	0.283	0.550	**0.040**	**0.015**
Hospitalization category	First episode (n=89)	20.2	4.5	9.0	2.2	3.4
	Recurrence (n=200)	17.5	7.5	11.5	5.0	9.0
	*χ^2^*	0.498	0.906	0.406	1.173	2.896
	*P*	0.480	0.341	0.524	0.279	0.089
Overweight / Obese	Yes (n=76)	34.2	13.2	11.8	11.8	18.4
	No (n=213)	9.4	4.2	4.7	1.4	3.3
	*χ^2^*	25.785	7.277	4.659	15.322	19.041
	*P*	**<0.001**	**0.007**	**0.031**	**<0.001**	**<0.001**
Aps	Monotherapy (n=171)	12.6	5.3	5.3	4.7	5.8
	Polypharmacy (n=118)	21.2	8.5	8.5	3.4	9.3
	*χ^2^*	6.524	5.898	5.898	0.291	1.251
	*P*	**0.023**	**0.015**	**0.015**	0.589	0.263

Ap, Antipsychotics; Bolded p value, < 0.05.

### 3.3 Analysis of independent risk factors for abnormal lipid levels

Variables demonstrating statistical significance in the univariate analysis were incorporated into a multivariate model. This analysis identified male sex (*OR* = 2.04, *P* = 0.016), overweight or obesity (*OR* = 4.55, *P* < 0.001), BRI (*OR* = 1.53, *P* = 0.005), and the combination of Aps (*OR* = 1.90, *P* = 0.030) as independent risk factors for dyslipidemia among EOS patients, as detailed in **Table 3**.

**Table 3:** Binary logistic regression analysis of dyslipidemia in EOS patients.

Variables.	*B*	*S.E.*	*P*	*OR*	Lower limit of *95.0% CI*	Upper limit of *95.0% CI*
Gender (ref: female)	0.713	0.297	**0.016**	2.04	1.14	3.65
Overweight / Obese (ref: normal)	1.515	0.303	**<0.001**	4.55	2.51	8.24
BRI	0.422	0.150	**0.005**	1.53	1.14	2.05
Polypharmacy (ref: Monotherapy)	0.640	0.294	**0.030**	1.90	1.07	3.38

BRI, Body Roundness Index; Bolded p value, < 0.05.

## 4 Discussion

This study revealed an aggregate dyslipidemia prevalence of 24.9%. Specifically, the rates for abnormal Triglycerides, Total Cholesterol, High-Density Lipoprotein Cholesterol, Low-Density Lipoprotein Cholesterol, and non-HDL-C were 15.9%, 6.6%, 6.6%, 4.2%, and 7.3%. According to the 2022–2023 national survey on nutrition and health among Chinese residents, 24.5% of children and adolescents exhibited dyslipidemia, which positively correlated with elevated risks of overweight or obesity in this demographic (16). A recent meta-analysis incorporating 134,438 subjects determined that the overall dyslipidemia prevalence in Chinese youth was 19%, with detection rates for elevated TG, high TC, reduced HDL-C, and increased LDL-C at 9%, 6%, 10%, and 4%, respectively (1). Thus, the prevalence of dyslipidemia in EOS patients was slightly higher than that in the general population. Clinical studies have found that even after short-term (4 weeks) administration of antipsychotic drugs, the patients’ body weight, TG, and LDL-C levels would significantly increase (17). For patients with early-onset mental disorders, various cardiovascular metabolic risk factors, such as obesity, hypertension, high TC, and low HDL-C, are closely related to their glycolipid metabolism disorders (18). A publication from the China CDC further underscored the importance of persistent surveillance of pediatric and adolescent lipid profiles, particularly triglycerides and high-density lipoprotein cholesterol. This monitoring is crucial for devising public health measures aimed at fostering sustained cardiovascular wellness and averting adult-onset cardiovascular conditions (19).

When comparing different groups, it was found that the abnormal rates of TG, LDL-C, and non-HDL-C in male patients were significantly higher than those in female patients. Previous studies also indicated the existence of gender differences. Jiang et al. observed a greater prevalence of dyslipidemia in overweight and obese male students compared to their female peers, with rates of 29.0% versus 18.9% respectively (20). Supporting this trend, research on 7–17-year-olds revealed that male participants had higher frequencies of elevated blood pressure, blood glucose, and total cholesterol. Moreover, boys in this age group exhibited a greater overall likelihood of presenting with cardiovascular metabolic risk factors (21). However, some studies showed different results. For instance, surveillance data on nutrition and health in western China from 2016 to 2017 revealed higher concentrations of several lipid markers and greater prevalence rates of elevated total cholesterol and low-density lipoprotein cholesterol among adolescent girls compared to boys. Conversely, boys exhibited a higher incidence of reduced high-density lipoprotein cholesterol (22). Such gender disparity might correlate with physiological variations in sex hormone concentrations occurring during pubertal development. Research has noted that males typically experience a swift decline in total cholesterol, low-density lipoprotein, and high-density lipoprotein levels in early puberty, whereas female levels tend to remain comparatively steady (23). Therefore, when formulating diagnostic criteria for dyslipidemia in children and adolescents, it is necessary to make more detailed divisions considering factors such as gender and age.

Furthermore, the prevalence of dyslipidemia, encompassing elevated TG, TC, LDL-C, and non-HDL-C levels, was markedly greater among overweight or obese subjects compared to their normal-weight counterparts. The respective incidence figures were 34.2% versus 9.1%, 13.2% versus 4.8%, 11.8% versus 1.6%, and 18.4% versus 3.8%. These data clearly indicate a substantially increased propensity for lipid metabolism abnormalities in individuals with excess body weight. The study further pointed out that abnormal lipid metabolism could increase the risk of overweight or obesity in children and adolescents (16), and lipid levels often increase stepwise with weight gain, with the order being the obese group > the overweight group > the normal weight group (22). Therefore, there is a bidirectional association of mutual promotion between lipid metabolism abnormalities and overweight or obesity.

The BRI index and the TyG index are two emerging metabolic assessment tools that hold significant value in the early screening and risk prediction of dyslipidemia in adolescents. The BRI utilizes anthropometric data, including height and waist circumference, to provide a more precise measure of visceral adiposity and central fat distribution. Research indicates this metric offers significant predictive value for identifying hypertension, dyslipidemia, and abdominal obesity across both genders (24). Additionally, as a comprehensive anthropometric indicator, the BRI demonstrates the best predictive ability for all cardiovascular metabolic abnormalities in females; while in males, it can more effectively predict hypertension and at least one cardiovascular metabolic abnormality (25). In contrast, the TyG index demonstrates a significant positive correlation with cardiovascular disease risk and is also associated with an elevated risk of all-cause mortality in populations, as indicated by studies (10, 26). This index exhibits a stable linear distribution characteristic by gender and age in children and adolescents, and can significantly predict the metabolic disorder state of the body (27). A potential mechanism involves elevated oxidative stress and damage to pancreatic β-cells, impairing insulin secretion and signal transduction. This dysregulation likely elevates the TyG index and subsequent cardiovascular risk (28). Notably, data show the TyG index is substantially higher in overweight/obese children compared to normal-weight peers and correlates positively with BMI, TC, and TG levels (29, 30). In summary, the BRI and the TyG index jointly reveal the pathophysiological mechanism of dyslipidemia from the perspectives of body fat distribution and metabolic disorder. Early monitoring of these two indicators helps identify adolescents at high risk of cardiovascular disease and provides a reference for disease prevention and intervention.

The administration of antipsychotic medications may induce dyslipidemia, which elevates patients’ cardiovascular disease risk (31). A four-week treatment with olanzapine or risperidone significantly raises serum concentrations of TG and LDL-C. This adverse metabolic effect occurs independently of any concurrent increase in body weight. They may persist even after adjusting for body mass index (32). Genetic factors also play a role in this process. Genome-wide analyses have revealed five specific single-nucleotide polymorphisms with significant links to medication-induced dyslipidemia, including: rs6532055 (LDL-C change caused by olanzapine), rs2644520 (TG change caused by aripiprazole), rs115843863 (HDL-C change caused by clozapine), rs2514895 (LDL-C change caused by paliperidone), and rs188405603 (TG change caused by quetiapine) (33). Follow-up studies have shown that TC, TG, and BMI significantly increase after 6 months of treatment; after continuous treatment for 3 years, FBG, insulin, TC, TG, insulin resistance index, and BMI all show a further upward trend (34). Collectively, these findings indicate that antipsychotic type, dose, and treatment duration exert cumulative adverse effects on lipid metabolism. Importantly, the present study identified combination therapy as an independent risk factor for dyslipidemia in EOS patients. This finding carries direct clinical implications: when clinically feasible, monotherapy should be prioritized to minimize metabolic risk. However, for patients who require combination therapy due to inadequate response or illness severity, more stringent and regular lipid monitoring is warranted, with proactive implementation of preventive interventions as clinically indicated.

In this study, the phenomenon that male patients with eosinophilia have a higher risk of dyslipidemia, apart from the hormonal changes during puberty, may also be related to schizophrenia and the gender-specific differences in the metabolism of antipsychotic drugs. Gender differences profoundly affect pharmacokinetics: women have less gastric acid and slower gastrointestinal emptying, which can alter drug absorption; a higher body fat percentage is prone to the accumulation of lipophilic drugs; the activity of liver CYP enzymes is regulated by estrogen, and the activity of CYP3A4 in women is higher than that in men while CYP1A2 is lower than that in men (35). Due to the metabolism of various atypical antipsychotic drugs through this pathway, the difference in enzyme activity may lead to gender differences in drug exposure and its metabolic effects (36). The interaction of these factors with the characteristics of the disease itself may partially explain the gender differences in the distribution of dyslipidemia in this study.

## 5 Limitations

This investigation acknowledges inherent limitations. Primarily, its cross-sectional design, while identifying correlates of various dyslipidemia subtypes in EOS, precludes definitive conclusions regarding causality between these markers and observed lipid profile deviations. Secondly, although the study compared the use of antipsychotic drugs alone or in combination and the drug dosage, the confounding effect of the drugs may lead to certain bias in the results. Finally, the study included only inpatients with EOS from a single center, thus, findings lack data from diverse geographic regions and varied ethnic populations, thereby limiting their generalizability. Future investigations involving multiple centers and larger samples are essential to enhance the broader applicability of these outcomes.

## 6 Conclusion

In conclusion, this study indicates that the prevalence of dyslipidemia in EOS patients is relatively high, and the distribution of single-category dyslipidemia varies significantly among different genders, body weight categories, and treatment regimens. Therefore, when clinicians pay attention to the lipid status of EOS patients, they should particularly focus on males, those who are overweight or obese, and individuals receiving combined drug treatment. Moreover, BRI and TyG index, as reliable metabolic assessment tools, are worthy of further in-depth exploration in the prediction and application of dyslipidemia in adolescents in the future.

## Data Availability

The datasets presented in this article are not readily available because The data used for this study are available from the corresponding author on reasonable request. Requests to access the datasets should be directed to Zhiwei Liu, zhiweiliu92@126.com.

## References

[1] Zhou Z Jia Y Yan H Xu J Wen J Wang S . Meta-analysis of the prevalence of dyslipidemia in Chinese children and adolescents. Chin Gen Pract. (2024) 27:2145–54.

[2] Correll CU Arango C Fagerlund B Galderisi S Kas M J Leucht S . Identification and treatment of individuals with childhood-onset and early-onset schizophrenia. Eur Neuropsychopharmacol. (2024) 82:57–71. doi: 10.1016/j.euroneuro.2024.02.005. PMID: 38492329

[3] Liu Z Xia L Zhang Y Wang J Zhong Y Liu H . Research progress on early symptoms and biomarkers of metabolic syndrome associated with antipsychotic drugs. J Int Psychiatry. (2019) 46:577–59. 85.

[4] Vochoskova K Mcwhinney SR Fialova M Kolenic M Spaniel F Svancer P . Weight and metabolic changes in early psychosis-association with daily quantification of medication exposure during the first hospitalization. Acta Psychiatr Scand. (2023) 148:265–76. doi: 10.1111/acps.13594. PMID: 37528692

[5] Nordentoft M Wahlbeck K Hällgren J Westman J Osby U Alinaghizadeh H . Excess mortality, causes of death and life expectancy in 270,770 patients with recent onset of mental disorders in Denmark, Finland and Sweden. PloS One. (2013) 8:e55176. doi: 10.1371/journal.pone.0055176. PMID: 23372832 PMC3555866

[6] Libowitz MR Nurmi EL . The burden of antipsychotic-induced weight gain and metabolic syndrome in children. Front Psychiatry. (2021) 12:623681. doi: 10.3389/fpsyt.2021.623681. PMID: 33776816 PMC7994286

[7] Ray WA Stein CM Murray KT Fuchs DC Patrick SW Daugherty J . Association of antipsychotic treatment with risk of unexpected death among children and youths. JAMA Psychiatry. (2019) 76:162–71. doi: 10.1001/jamapsychiatry.2018.3421. PMID: 30540347 PMC6440238

[8] Lin H Jia X Yin Y Li M Zheng R Xu Y . Association of body roundness index with cardiovascular disease and all-cause mortality among Chinese adults. Diabetes Obes Metab. (2025). 27(5):2698–2707. doi: 10.1111/dom.16272. PMID: 39972403

[9] Zhang F Shi W Wen J Cao H Xu W Lan T . Elevated body roundness index increases the risk of cardiovascular disease in Chinese patients with circadian syndrome. Front Endocrinol (Lausanne). (2025) 16:1532344. doi: 10.3389/fendo.2025.1532344. PMID: 40070588 PMC11893425

[10] Kwak J Han KD Lee EY Lee SH Lim DJ Kwon HS . Association between the triglyceride-glucose index and cardiovascular risk and mortality across different diabetes durations: a nationwide cohort study. Endocrinol Metab (Seoul). (2025). 40(4):548–560. doi: 10.3803/enm.2024.2205. PMID: 40040387 PMC12409153

[11] Leucht S Samara M Heres S Davis JM . Dose equivalents for antipsychotic drugs: the DDD method. Schizophr Bull. (2016) 42 Suppl 1:S90–4. doi: 10.1093/schbul/sbv037. PMID: 27460622 PMC4960429

[12] Imre O Imre G Mustu M Acat O Kocabas R . Cardiovascular disease markers in schizophrenia during negative symptoms and remission periods. J Clin Med. (2025) 14(7):2288. doi: 10.3390/jcm14072288. PMID: 40217736 PMC11989828

[13] Zhang L Yin J Sun H Dong W Liu Z Yang J . The relationship between body roundness index and depression: a cross-sectional study using data from the National Health and Nutrition Examination Survey (NHANES) 2011–2018. J Affect Disord. (2024) 361:17–23. doi: 10.1016/j.jad.2024.05.153. PMID: 38815765

[14] Lin CH Lin HS Lin SC Kuo CC Wang FC Huang YH . Early improvement in PANSS-30, PANSS-8, and PANSS-6 scores predicts ultimate response and remission during acute treatment of schizophrenia. Acta Psychiatr Scand. (2018) 137:98–108. doi: 10.1111/acps.12849. PMID: 29280500

[15] Wang Z Liu J Li J Wu N Lu G Chen Z . Chinese guidelines for lipid management (2023). Chin Circ J. (2023) 38:1190934. doi: 10.3389/fphar.2023.1190934. PMID: 37711173 PMC10498001

[16] Li T Luo X Tian M Liu C . The association between the interaction of vitamin D deficiency and dyslipidemia and the risk of overweight and obesity in children and adolescents. Chin J Sch Health. (2024) 45:1378–82. doi: 10.34297/ajbsr.2021.11.001673

[17] Shu C Wang X Liu J . The effects of atypical antipsychotic drugs on metabolism in adolescent schizophrenia patients of different genders. J Neural Inj Funct Reconstr. (2018) 13:508–10.

[18] Smith J Griffiths LA Band M Horne D . Cardiometabolic risk in first episode psychosis patients. Front Endocrinol (Lausanne). (2020) 11:564240. doi: 10.3389/fendo.2020.564240. PMID: 33329382 PMC7732528

[19] Deng X Zhang M Zhang X Huang Z Zhao Z Li C . The status of blood lipids among children and adolescents - China, 2016–2017. China CDC Weekly. (2023) 5:31–4. doi: 10.46234/ccdcw2023.006. PMID: 36776688 PMC9902742

[20] Jiang N Yu H Wang S . Investigation on dyslipidemia in overweight and obese primary and secondary school students in Tongzhou District. J Prev Med. (2022) 34:87–90.

[21] Hu X Jiang H Zhang B Wang H Zhang J Jia X . Prevalence characteristics of cardiovascular and metabolic risk factors among children and adolescents aged 7–17 years in 15 provinces of China. J Environ Occup Med. (2021) 38:833–8. doi: 10.26599/1671-5411.2024.02.008. PMID: 38544492 PMC10964013

[22] Li Y Jia S Liu B Fang H Ding X Zhang J . Analysis of the prevalence of dyslipidemia among children aged 12–17 in western China in 2016–2017. Chin J Public Health. (2021) 37:1508–13.

[23] Laskarzewski PM Morrison JA Gutai J Khoury PR Glueck CJ . Longitudinal relationships among endogenous testosterone, estradiol, and Quetelet index with high and low density lipoprotein cholesterols in adolescent boys. Pediatr Res. (1983) 17:689–98. doi: 10.1203/00006450-198308000-00018. PMID: 6889012

[24] Chen R Ji L Chen Y Meng L . Weight-to-height ratio and body roundness index are superior indicators to assess cardio-metabolic risks in Chinese children and adolescents: compared with body mass index and a body shape index. Transl Pediatr. (2022) 11:318–29. doi: 10.21037/tp-21-479. PMID: 35378962 PMC8976678

[25] Tian S Zhang X Xu Y Dong H . Feasibility of body roundness index for identifying a clustering of cardiometabolic abnormalities compared to BMI, waist circumference and other anthropometric indices: the China Health and Nutrition Survey, 2008 to 2009. Med (Baltimore). (2016) 95:e4642. doi: 10.1097/md.0000000000004642. PMID: 27559964 PMC5400331

[26] Feng Y Yin L Huang H Hu Y Lin S . Assessing the impact of insulin resistance trajectories on cardiovascular disease risk using longitudinal targeted maximum likelihood estimation. Cardiovasc Diabetol. (2025) 24:112. doi: 10.1186/s12933-025-02651-6. PMID: 40065358 PMC11895167

[27] Yoon JS Shim YS Lee HS Hwang IT Hwang JS . A population-based study of TyG index distribution and its relationship to cardiometabolic risk factors in children and adolescents. Sci Rep. (2021) 11:23660. doi: 10.1038/s41598-021-03138-6. PMID: 34880367 PMC8654923

[28] Sardar MB Raza M Fayyaz A Nadir MA Nadeem ZA Babar M . Environmental heavy metal exposure and associated cardiovascular diseases in light of the triglyceride glucose index. Cardiovasc Toxicol. (2024) 24:1301–9. doi: 10.1007/s12012-024-09913-x. PMID: 39212843

[29] Dikaiakou E Vlachopapadopoulou EA Paschou SA Athanasouli F Panagiotopoulos Ι Kafetzi M . Triglycerides-glucose (TyG) index is a sensitive marker of insulin resistance in Greek children and adolescents. Endocrine. (2020) 70:58–64. doi: 10.1007/s12020-020-02374-6. PMID: 32557329

[30] Yoon JS Lee HJ Jeong HR Shim YS Kang MJ Hwang IT . Triglyceride glucose index is superior biomarker for predicting type 2 diabetes mellitus in children and adolescents. Endocr J. (2022) 69:559–65. doi: 10.1507/endocrj.ej21-0560. PMID: 34924455

[31] Pereira S Au E Agarwal SM Wright DC Hahn MK . Antipsychotic-induced alterations in lipid turnover. Endocrinology. (2023) 164:bqad025. doi: 10.1210/endocr/bqad025. PMID: 36718081

[32] Yang Y Wu R . Atypical antipsychotic drugs cause abnormal glucose and lipid metabolism independent of weight gain. Eur Arch Psychiatry Clin Neurosci. (2025) 275:619–27. doi: 10.1007/s00406-025-01965-6. PMID: 39969542

[33] Wong KC Leung PB Lee BK Zheng ZZ Tsang EM Liu MH . Pharmacogenetic study of antipsychotic-induced lipid and BMI changes in Chinese schizophrenia patients: a genome-wide association study. Transl Psychiatry. (2025) 15:295. doi: 10.1101/2024.09.04.24313052. PMID: 40830362 PMC12365156

[34] Petrikis P Tigas S Tzallas AT Ntritsos G Sioka C Georgiou G . Effects of antipsychotic medications in glucose and lipid metabolism at the fasted state in drug-naïve first episode patients with psychosis after six months and three years of treatment. Psychiatriki. (2024) 35:187–98. doi: 10.22365/jpsych.2024.008. PMID: 38814269

[35] Sommer IE Brand BA Stuijt CCM Touw DJ . Sex differences need to be considered when treating women with psychotropic drugs. World Psychiatry. (2024) 23:151–2. doi: 10.1002/wps.21155. PMID: 38214636 PMC10785976

[36] Brissos S Balanzá-Martínez V . Long-acting antipsychotic treatments: focus on women with schizophrenia. Ther Adv Psychopharmacol. (2024) 14:20451253241263715. doi: 10.1177/20451253241263715. PMID: 39091697 PMC11292690

